# Prevention of Calcium Hydroxyapatite Nodules in the Neck With Use of Botulinum Toxin: A Case Report and Review of Anatomy and Pathophysiology

**DOI:** 10.1111/jocd.70385

**Published:** 2025-08-09

**Authors:** Bianca Y. Kang, Sabrina Guillen Fabi, Elika Hoss

**Affiliations:** ^1^ Department of Dermatology Mayo Clinic Scottsdale Arizona USA; ^2^ Cosmetic Laser Dermatology San Diego California USA; ^3^ Department of Dermatology University of California San Diego San Diego California USA

**Keywords:** botulinum toxin, calcium hydroxyapatite, complications, cosmetic dermatology, filler, neck

## Abstract

**Background:**

Calcium hydroxylapatite (CaHA) is a biostimulatory filler frequently used in a hyperdilute form to improve skin quality. While generally safe, nodule formation is the most common adverse event, particularly in dynamic areas.

**Aims:**

To describe a case of delayed‐onset CaHA nodules in the neck and review anatomical and procedural factors contributing to this complication, as well as prevention and management strategies.

**Patient:**

A 72‐year‐old woman underwent three sessions of hyperdilute CaHA injections to the neck, with recent or concurrent onabotulinumtoxinA injections. After the third treatment, which followed a longer interval since her last botulinum toxin injection, she developed multiple firm, non‐tender nodules. A stepwise approach with intralesional saline injections—initially using needles and later a cannula—was implemented.

**Results:**

Initial saline injections with small‐gauge needles yielded minimal improvement. Transitioning to a 22G cannula enabled both saline delivery and mechanical disruption, resulting in complete resolution after five total treatments. The patient has remained recurrence‐free for over 2 years.

**Conclusions:**

Nodule formation following CaHA injection is most often due to product aggregation, and is particularly common in dynamic areas with thin overlying skin. Combining treatment with botulinum toxin may reduce this risk by limiting movement. Other preventive strategies include using higher dilutions of CaHA, avoiding high‐risk areas, and optimizing injection depth. When nodules do occur, mechanical disruption with a cannula combined with saline injection is a safe, effective, and minimally invasive first‐line treatment.

## 1 Background

Radiesse (Merz Aesthetics, Raleigh, NC) is a biostimulatory filler containing calcium hydroxylapatite (CaHA) microspheres, which stimulate collagen and elastin production [1]. Hyperdilute CaHA (≥ 1:1 dilution) injection is an off‐label technique commonly used on the face and body to improve skin quality and firmness [1]. Although uncommon, nodules are the most frequent adverse event. We report a case of non‐tender nodules on the lower neck following CaHA injection, and we review contributing factors and strategies for prevention.

## 2 Case Report

A 72‐year‐old woman presented for treatment of neck aging. Examination showed crepiness and platysmal banding. Combined treatment with hyperdilute CaHA and Botox (onabotulinumtoxinA) was recommended.

The first two treatments, spaced 3 months apart, each involved one syringe of CaHA filler diluted 1:3 with bacteriostatic saline and injected using a 22G cannula in a retrograde threading pattern in the subdermal plane bilaterally. Local infiltration of lidocaine was used at cannula insertion sites, and treated areas were massaged postinjection. Both treatments were combined with onabotulinumtoxinA (Botox, Abbvie, Irvine, CA) injections to the platysma, either at the same visit or 2 weeks prior, and resulted in satisfactory outcomes without adverse events.

**FIGURE 1:**
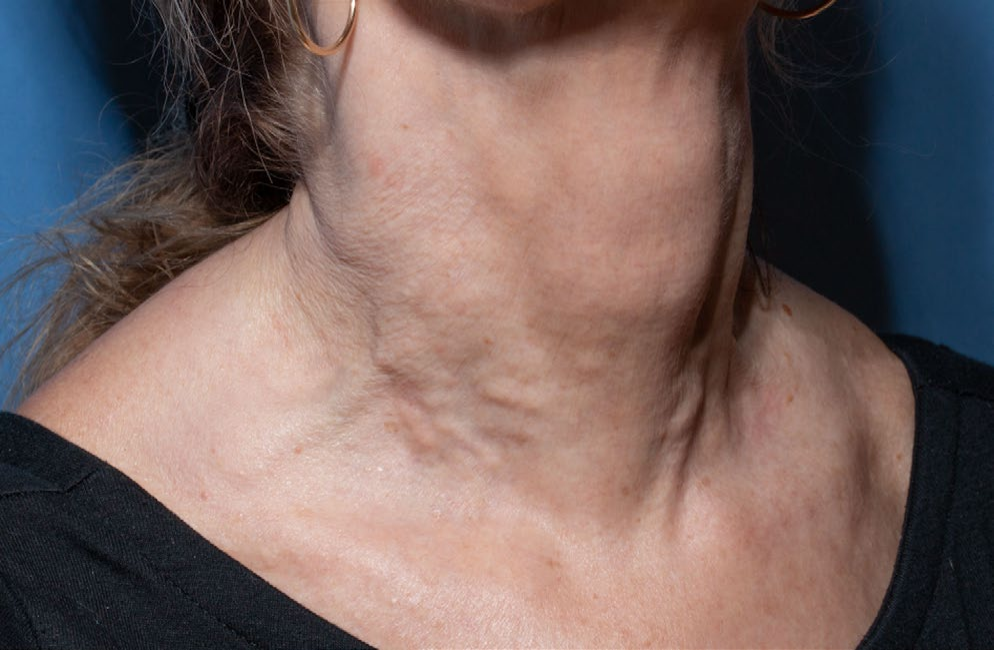
Nodules following injection of calcium hydroxylapatite to the lower neck. Image taken approximately 3 months after most recent injection.

Nine months later, the patient underwent a third CaHA treatment using the same injection technique. However, her most recent botulinum toxin injection had been 2 months earlier. Approximately 3 months after this session, she noted multiple firm, non‐tender nodules on the lower neck (Figure 1).

Due to the lack of standardized guidelines for this complication, we initiated intralesional saline injections and modified our approach based on response. Other treatments, including corticosteroids and 5‐fluorouracil, were considered but declined by the patient in favor of a low‐risk, minimally invasive approach. Injections were performed at 2‐week intervals. Saline was chosen based on prior studies suggesting in situ hyperdilution with saline and massage as a first‐line strategy [2, 3], which is believed to disperse CaHA particles and promote clearance of aggregated microspheres.

**FIGURE 2:**
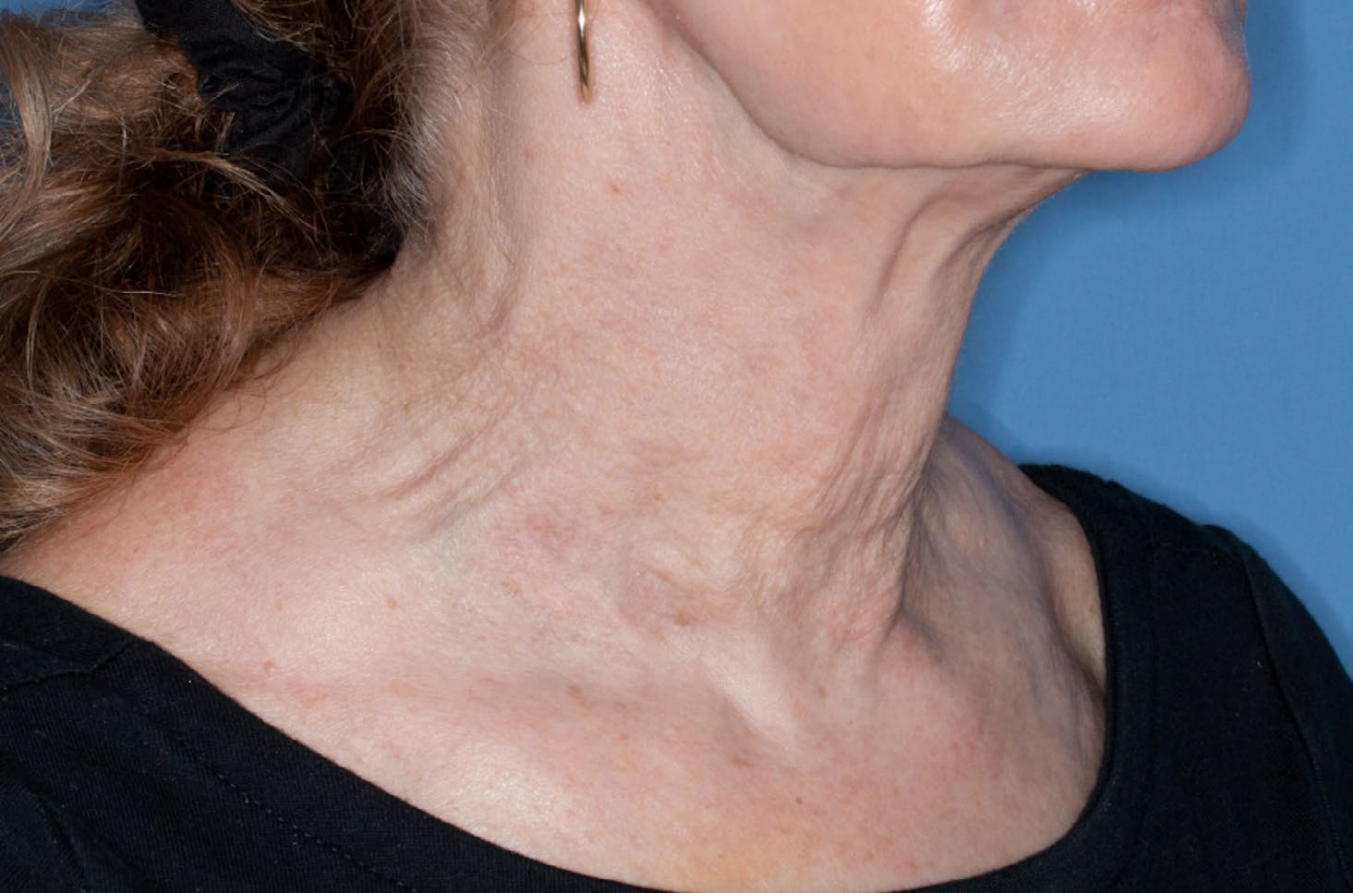
Resolution of nodules after five saline injections over a 10‐week period.

The initial treatment used a 30G needle followed by massage, resulting in minimal improvement. A 27G needle was used for the second injection, again followed by massage, still resulting in only mild improvement. Subsequent treatments were performed with a 22G cannula, which not only allowed for saline injection but also provided additional mechanical disruption of the nodules, simulating a subcision‐like effect. The clinical endpoint for each session was complete saline infiltration extending approximately 5 mm beyond each nodule. On average, 2.5 cc of saline was used per nodule. This approach led to incremental improvement and ultimately complete resolution after five total saline injections (three with a cannula) (Figure 2). The patient has continued regular follow‐up for over 2 years without recurrence of nodules.

## 3 Discussion

Although rare, nodule formation is the most frequently reported complication of CaHA filler injection, occurring after approximately 3% of injections [4]. Due to the absence of standardized treatment protocols, various strategies have been described in the literature. Understanding the pathogenesis and contributing factors is key to prevention and management.

CaHA nodules most often result from product aggregation, either due to muscle activity, injection technique (e.g., superficial injection, overfilling, and insufficient dilution), or, in rare cases, filler migration [2, 4, 5]. Among the known contributing factors, repeated muscular contraction leading to microsphere aggregation is believed to be the most common cause of CaHA nodules overall [4]. In a review of 5081 CaHA injections, 166 nodules were reported, most commonly at the lips (45%), perioral region (4%), and nasolabial folds (3%), highlighting a predilection for dynamic facial areas [4]. Less frequently, nodules may result from hypersensitivity reactions, infection, biofilm formation, or granulomatous reaction [2, 4]. Various classification systems have categorized CaHA nodules based on clinical features, including inflammatory versus noninflammatory and early versus delayed onset [2, 4]. These classifications can be useful in guiding differential diagnosis and treatment selection, particularly when symptoms such as erythema, tenderness, or rapid onset are present. In clinical practice, however, the majority of CaHA nodules are due to product aggregation, so it is reasonable to initially assume a mechanical etiology and pursue standard dispersion treatments. Alternative diagnoses should be considered if nodules are refractory or if there are signs or symptoms concerning for infection.

In the authors' experience, combined treatment with neuromodulator appears to reduce nodule risk by limiting muscular contraction. Botulinum toxin reaches peak effect at 4 weeks and wanes thereafter, potentially explaining reduced protection during the third injection in this case report [6]. Studies with hyaluronic acid (HA) filler show that combining with botulinum toxin can extend filler longevity by reducing muscle‐driven degradation [7, 8, 9, 10]. With biostimulatory fillers like CaHA, reduced muscle activity may instead prevent particle aggregation by minimizing repetitive mechanical shear forces in dynamic areas. Although direct evidence for this mechanism with CaHA is limited, the observed pattern of nodule development in high‐mobility areas supports this hypothesis and has informed expert recommendations for using neuromodulators as a preventive strategy.

Injection depth is also critical, especially because many patients who are good candidates for hyperdilute CaHA also have significant photoaging‐related dermal and epidermal atrophy. The platysma in the lower neck is more superficial (frequently 0.75–3 mm beneath the skin surface), due to thin skin and frequent adhesions between the skin and the underlying platysma. This varies depending on patient factors such as age, body mass index (BMI), and sex [11]. Because of this, more superficial injections (into the subdermal plane) may reduce the risk of CaHA accumulation within or below the platysma. In our current clinical practice, we also recommend avoiding injection into the lower 20% of the neck (Table 1).

**TABLE 1:** Clinical pearls for calcium hydroxylapatite injections in the neck.

Clinical pearls for calcium hydroxylapatite injections in the neck
Administer botulinum toxin 2–4 weeks prior to CaHA injection to reduce platysmal movement and lower the risk of product aggregation. If onabotulinumtoxinA is used, the FDA‐approved dosing for the neck is 26–36 units. Avoid or use caution when injecting into the lower 20% of the neck, where the platysma lies more superficially and the skin is thinner, increasing the risk of superficial filler accumulation. Use higher dilutions of CaHA (e.g., 1:2–1:4) when treating thin or atrophic neck skin to minimize the risk of nodule formation. Limit CaHA volume to one syringe per session and adopt a slow, gradual approach to treatment. Injecting with a cannula or using a short linear threading technique with a needle may help to minimize trauma and improve control. If nodules develop and treatment is desired, initiate first‐line management with saline injection and massage. Cannula‐based saline infiltration may be more effective due to its ability to simultaneously deliver fluid and mechanically disrupt aggregates. Consider alternative diagnoses (e.g., infection, biofilm, and granulomatous reaction) and escalate to second‐ or third‐line therapies (e.g., intralesional corticosteroids or 5‐FU, fractional ablative laser, or surgical excision) only if nodules persist despite standard dispersion techniques.

Abbreviations: 5‐FU, 5‐fluorouracil; CaHA, calcium hydroxylapatite; FDA, US Food and Drug Administration.

Dilution is another important consideration. The current consensus for treatment of the neck with hyperdilute CaHA is to use higher dilutions (1:2–1:4, depending on skin thickness), as these are thought to carry a lower risk of nodule development [1]. Additional recommended techniques to prevent CaHA nodules are treating gradually, limiting each session to one syringe of product, and injecting with a cannula [1]. A short linear threading technique with a needle is an alternative technique. As demonstrated in this case, these techniques alone were insufficient in preventing nodule formation when platysmal activity was present.

When nodules do develop in any location, a stepwise approach to treatment is advised. Observation is an option, particularly for inconspicuous nodules. If treatment is desired, early intervention is key, as prolonged CaHA‐stimulated collagen and elastin production may increase the difficulty of treatment overtime [2, 4]. Generally, initial management involves dispersion techniques, such as saline or sterile water injections followed by massage [2, 4]. Our findings suggest that simple saline injections using small‐gauge needles provide only mild improvement, whereas cannula‐based injections offer superior efficacy by simultaneously delivering saline while mechanically disrupting the nodules. If nodules persist, second‐line options include intralesional injections of 5‐fluorouracil and/or corticosteroids, laser therapy, or surgical excision (Table 2).

**TABLE 2:** Stepwise approach to managing calcium hydroxylapatite nodules, adapted from McCarthy et al. [2].

Approach	Modality	Mechanism	Clinical considerations
No treatment	Observation	Natural degradation of CaHA over time	Suitable for asymptomatic, non‐visible nodules; based on patient preference
First line: minimal intervention	Aqueous dispersion (saline injections and massage)	Mechanical dispersion of CaHA particles	Common approach; effective for early or mild nodules; low‐risk; consider cannula rather than needle
	Other forms of mechanical dispersion (e.g., radiofrequency, mechanized microneedling, thermomechanical ablation)	Mechanical dispersion of CaHA particles	Requires specialized equipment; emerging technique
Second line: pharmacological and laser interventions	Intralesional corticosteroids (e.g., triamcinolone, dexamethasone)	Anti‐inflammatory; reduces collagen production	Risks include skin atrophy, pigmentation changes; most useful for inflammatory nodules
	Intralesional 5‐fluorouracil (5‐FU)	Antimetabolite; inhibits fibroblast proliferation	May be combined with corticosteroids; consider for persistent nodules
	Fractional ablative laser therapy (CO_2_ or Er:YAG)	Thermal ablation; promotes remodeling	Best for superficial or fibrotic nodules; risk of pigmentation changes
	Ultrasound delivery of collagenase	Enhances enzymatic breakdown of CaHA aggregates	Investigational; safety not established
Third line: invasive removal	Incision/lancing and drainage	Physical removal of the nodule	Invasive with potential for scarring
	Surgical excision	Physical removal of the nodule	Invasive with potential for scarring
	Negative pressure microcannula aspiration	Physical removal of the nodule	Invasive with potential for scarring

Abbreviation: CaHA, calcium hydroxylapatite.

## 4 Conclusion

Hyperdilute CaHA is an effective treatment for improving skin quality and firmness. While nodule formation is uncommon, it remains the primary adverse event, especially in dynamic areas with thin overlying skin. This case highlights preventive strategies, including injection technique, product dilution, and adjunctive use of neuromodulators. When nodules do occur, mechanical disruption with a cannula combined with saline injection is a safe and effective first‐line treatment. By continuing to refine injection strategies and improving our understanding of nodule formation, we can improve safety and efficacy in aesthetic treatments with CaHA.

## Disclosure

Dr. Fabi is a consultant for AbbVie, Galderma, Merz, Revance, L'Oréal; investigator for AbbVie, Galderma, Merz, Revance, Croma, Symatese, L'Oréal; and stockholder in Revance and AbbVie. Dr. Kang and Dr. Hoss have no relevant disclosures.

## Ethics Statement

This case report did not require institutional review board (IRB) approval as it presents a clinical case for educational purposes only, with all patient identifying information de‐identified in accordance with HIPAA regulations. Written, informed consent was obtained from the patient for all procedures performed.

## Consent

Written, informed consent was obtained from the patient for publication of this case report and accompanying images.

## Conflicts of Interest

The authors declare no conflicts of interest.

## Data Availability

Data sharing not applicable to this article as no datasets were generated or analysed during the current study.
